# Equivariant Graph Neural Networks for Toxicity Prediction

**DOI:** 10.1021/acs.chemrestox.3c00032

**Published:** 2023-09-10

**Authors:** Julian Cremer, Leonardo Medrano Sandonas, Alexandre Tkatchenko, Djork-Arné Clevert, Gianni De Fabritiis

**Affiliations:** † Computational Science Laboratory, Universitat Pompeu Fabra, Barcelona Biomedical Research Park (PRBB), Carrer Dr. Aiguader 88, 08003 Barcelona, Spain; ‡ Machine Learning Research, Pfizer Worldwide Research Development and Medical, Linkstr. 10, 10785 Berlin, Germany; § Department of Physics and Materials Science, University of Luxembourg, L-1511 Luxembourg City, Luxembourg; ⊥ ICREA, Passeig Lluis Companys 23, 08010 Barcelona, Spain

## Abstract

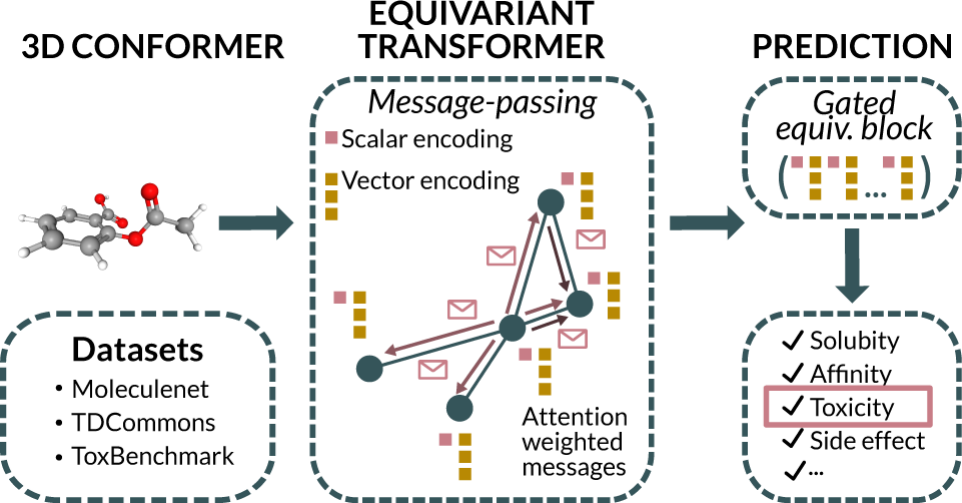

Predictive modeling of toxicity is a crucial step in the drug discovery pipeline. It can help filter out molecules with a high probability of failing in the early stages of de novo drug design. Thus, several machine learning (ML) models have been developed to predict the toxicity of molecules by combining classical ML techniques or deep neural networks with well-known molecular representations such as fingerprints or 2D graphs. But the more natural, accurate representation of molecules is expected to be defined in physical 3D space like in ab initio methods. Recent studies successfully used equivariant graph neural networks (EGNNs) for representation learning based on 3D structures to predict quantum-mechanical properties of molecules. Inspired by this, we investigated the performance of EGNNs to construct reliable ML models for toxicity prediction. We used the equivariant transformer (ET) model in TorchMD-NET for this. Eleven toxicity data sets taken from MoleculeNet, TDCommons, and ToxBenchmark have been considered to evaluate the capability of ET for toxicity prediction. Our results show that ET adequately learns 3D representations of molecules that can successfully correlate with toxicity activity, achieving good accuracies on most data sets comparable to state-of-the-art models. We also test a physicochemical property, namely, the total energy of a molecule, to inform the toxicity prediction with a physical prior. However, our work suggests that these two properties can not be related. We also provide an attention weight analysis for helping to understand the toxicity prediction in 3D space and thus increase the explainability of the ML model. In summary, our findings offer promising insights considering 3D geometry information via EGNNs and provide a straightforward way to integrate molecular conformers into ML-based pipelines for predicting and investigating toxicity prediction in physical space. We expect that in the future, especially for larger, more diverse data sets, EGNNs will be an essential tool in this domain.

## Introduction

Next to the tremendous success of machine learning (ML) in computer vision and language processing, ML has emerged as a promising tool in many research fields in natural sciences like physics, chemistry, and biology.

Specifically, ML has been successfully applied to investigate and predict the quantitative structure–activity relationship (QSAR), which is one of the most critical tasks in computational drug and material discovery, as many methods downstream rely on accurate molecular activity predictions for evaluating, selecting, or even generating new molecules.^1−4^ QSAR modeling is a computational technique that uses mathematical and statistical methods to model the relationship between biological or physicochemical endpoints and the structural characteristics of chemical compounds. This allows researchers to rapidly evaluate the potential usefulness of large numbers of compounds, saving time and resources in the notoriously long drug discovery process.^5,6^ QSAR modeling became closely related to machine learning, as ML is predestined as a toolbox to predict structure–activity relationships based on supervised training. In QSAR modeling, the input to the ML algorithm is the structural characteristics of a compound, and the output is a prediction of its ADMET profile.^7,8^ One biological endpoint is usually the binding affinity of a drug candidate against a protein target. Because drug candidates with high binding affinity can still fail in later phases of clinical trials due to poor pharmacokinetic and toxicological profiles, modeling different ADMET endpoints such as solubility, melting point, or toxicity, is nowadays also considered in in silico de novo drug design at early stages.

However, conventional ML approaches for QSAR modeling have mainly focused so far on feature engineering for molecular descriptors based on fingerprint-, InChi-, SMILES-, or 2D-graph-based molecular representations.^6,8−11^ Besides the remarkable results of QSAR models in the past side by side with the improvements in virtual screening, there is evidence that the 3D structure of the molecules can significantly influence physical, chemical, and biological activity.^4,12,13^ For instance, cis-Platin is used as a chemotherapy drug, whereas its stereoisomer, trans-Platin, does not show cytotoxic activity.^4^ Nevertheless, existing representation methods mainly encode the topological information on molecules rather than the molecular geometry information, and consequently, they lack the ability to distinguish between molecules with the same topology but different 3D geometry (e.g., stereoisomerism) as the example of cis- and trans-Platin clearly shows. In addition, based on well-known quantum-mechanical methods, to achieve ab initio performance and thus the most accurate property calculation, it seems imperative to change the perspective to a more precise modeling and representation learning of molecules in 3D space. Recent work by Fang et al.^4^ introduces 3D spatial knowledge by using bond-angle and atom-bond graphs, but the authors do not directly incorporate the 3D structure of the molecules and do not make use of equivariant atomic embeddings. Equivariance in this context is restricted to isometries of the Euclidean space, namely, global rotations, and translations, which cover the main symmetries in molecular systems. Hence, the effect of integrating adequate 3D molecular representations into equivariant ML-based frameworks for toxicity prediction is an open question that still needs further examination.

Prominent supervised examples encoding 3D information in the form of molecular conformations are neural network potentials (NNPs) and ML force fields (MLFFs), as well as ML models that predict a plethora of physical, chemical, and biological activities/properties.^4,8,14−26^ NNPs and MLFFs have been trained using a data set of known atomic or molecular structures and their corresponding energies and atomic forces. This allows researchers to access accurate and fast calculations of the potential energy surfaces and physicochemical properties of complex systems such as proteins and materials.^15,16,27−31^ By predicting the energy of different protein structures, NNPs can be used to identify a protein’s most stable and functional conformation, a subject known as the protein folding problem.^32,33^

Crucially, the performance of such ML models has proven to be significantly dependent on architectural choices. Graph-neural networks (GNNs), in contrast to any hand-crafted, fingerprint- or SMILES-based representations, can model the complex interactions between atoms end-to-end based only on the respective atomic coordinates and atom types. The learning algorithm for 3D GNNs is most often constructed as a message-passing neural network (MPNN) based on a 3D graph built by molecular geometry, with nodes being the atom types and edges being the relative distances between atoms. Here, high-dimensional atomic representations are refined by a message-passing scheme on the graph and can then be used for predicting specific atomic and molecular properties.^15,16,28^ Notably, incorporating physical inductive biases into the model by either restricting the input space accordingly or the mechanics of the model itself, like in the form of energy conservation and rotation, translation, and permutation equivariance, has proven to be key for the recent success. Consequently, modern GNN/MPNN-based models have emerged as a new paradigm to build powerful molecular and atomic representations. Prominent examples are SchNet,^15^ PaiNN,^16^ DimeNet,^34^ GemNet,^35^ TorchMD-NET,^28^ SpookyNet,^17^ NequIP,^29^ and MACE.^36^ Using these physics-inspired neural networks for toxicity prediction would not only answer the open question formulated above but also allow us to gain more insights into developing reliable toxicity predictive models.

**Figure 1:**
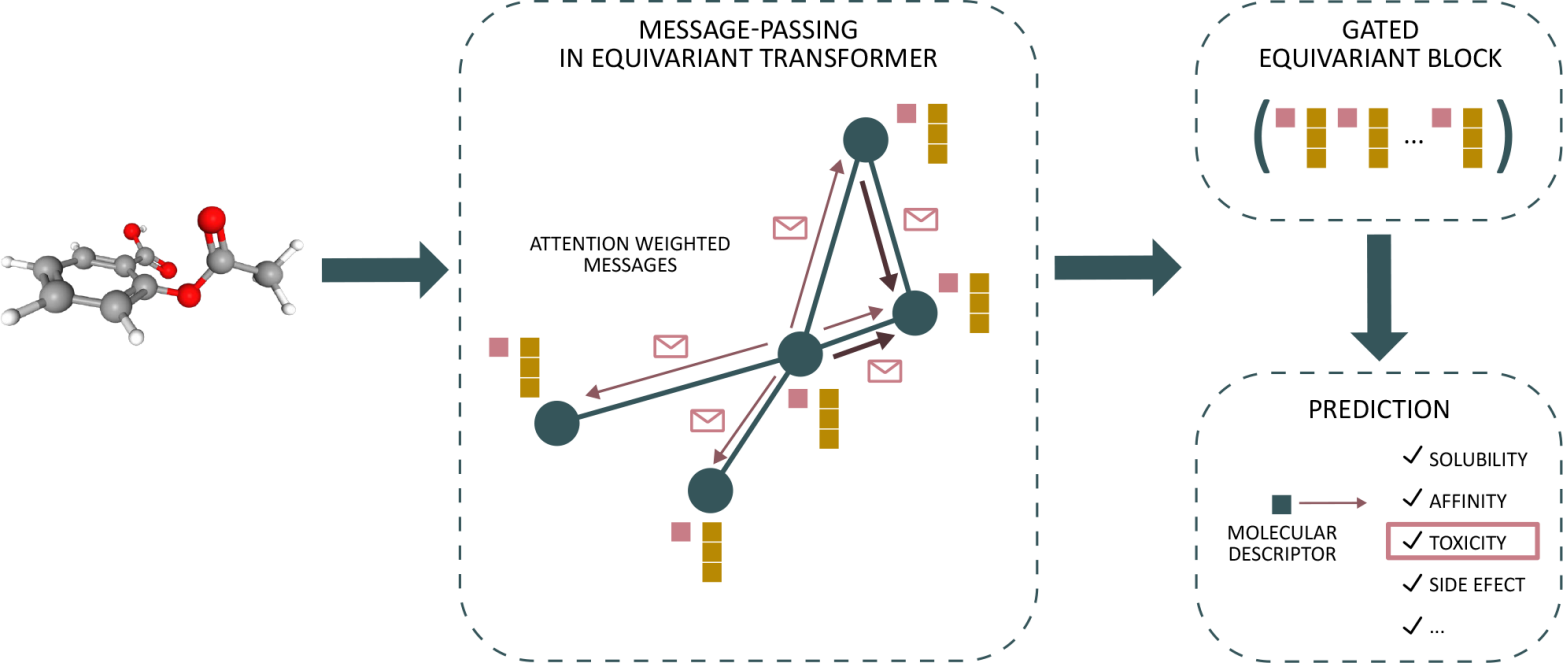
Overview of the method used in this work. An equivariant transformer graph neural network^28^ is applied to 3D conformers for toxicity prediction. The model uses scalar (pink) as well as vector features (brown) as node embeddings for messages in every message-passing step. A gated equivariant block^16^ combines both scalar and vector features and outputs a molecular descriptor that here is used to predict toxicity.

To address this challenge, in this study, we evaluate the performance of an equivariant graph neural network (EGNN) TorchMD-NET^28^ to generate adequate atomic and molecular structure representations for QSAR modeling. The workflow is depicted in Figure 1. To the best of our knowledge, this is the first work that thoroughly explores the capability of EGNNs in toxicity prediction based on only the geometry of high-quality 3D conformers. In doing so, the representational capacities of the equivariant transformer (ET) and a SMILES-based transformer were evaluated and quantified on the MoleculeNet,^3^ ToxBenchmark,^37^ as well as Therapeutic Data Commons (TDCommons)^38^ data sets. We used the 3D conformations provided in the GEOM^39^ data set for training on physiology/toxicity-related tasks of MoleculeNet. However, high-quality 3D-conformers data sets were generated for the ToxBenchmark and TDCommons data sets using the conformational search workflow implemented in CREST(40) that uses the semiempirical method GFN2-xTB.^41^ Our results set new benchmarks on the MoleculeNet data set using the 3D-conformers taken from the GEOM^39^ data set. The role of an additional molecular feature such as total energy when predicting toxicity has also been studied, yielding negative performance that reveals a lack of correlation between energetics and toxicity activity. Moreover, an exhaustive examination of the correlation between 3D structure and 12 biological properties (Tox21) demonstrated that ET considerably outperforms the SMILES transformer model for all tasks. We concluded this study with a comprehensive statistical analysis of the attention weights generated by the trained models on test set molecules, allowing us to have a better interpretation of our findings. Hence, we expect our results to provide novel insights that enable more accurate structure–activity relationships to improve toxicity prediction.

## Computational Methods

### Graph and Message-Passing Neural Networks

Graph neural networks (GNNs) are a type of ML algorithm that is designed to operate on data represented as a graph, a mathematical structure consisting of a set of vertices (or nodes) and a set of edges that connect the vertices. GNNs are particularly well-suited for modeling complex, interconnected data, such as the connectivity of social networks or the structure of molecules.^42,43^ On the other hand, message-passing neural networks (MPNNs) are a type of GNN that operate by passing messages between the nodes in a graph. In an MPNN, each node in the graph is associated with a neural network, and the edges of the graph define how messages are passed between these neural networks.^44^ Here,  is the graph where  is the set of nodes and  is the set of edges. Then the operation of an MPNN can be defined by the following equations:
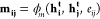
1
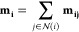
2
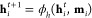
3with **m**_**ij**_ as the message between node *i* and node *j* with edge attributes *e*_*ij*_, **m**_*i*_ the aggregated message of all neighbors of node *i* (), and  being the hidden state of node *i* with h-dimensional embedding at layer *t. ϕ*_*m*,*h*_ are node and edge operations normally parametrized by multilayer perceptrons (MLPs) on messages and node updates, respectively.^42,45^

### Equivariant Transformer

Equivariance is a property of certain mathematical models and algorithms that ensures that the output of the model or algorithm is the same as that if the input had been transformed in a particular way. In the context of 3D graph neural networks, equivariance is crucial because it allows the network to process 3D data that have been transformed in various ways such as through rotation or translation of the data. In the context of molecular modeling, rotations as well as translations of a molecule do not change its scalar properties, but vectorial and tensorial properties need to change accordingly.

Mathematically, the equivariance can be described as follows. Let *f*: *X* → *Y* be a function that takes an input **x**. Let *T*_g_: *X* → *X* be a set of transformations on *X* for abstract group *g* ∈ *G*. If *f* is equivariant with respect to *g* given a transformation on the output space *S*_*g*_: *Y* → *Y*, then we have the following relationship:
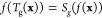
4

This equation states that if we apply transformation *g* to the input of the function *f*, the resulting output is the same as if we had applied transformation *g* to the output of function *f*. EGNNs used in neural network potentials focus on equivariance under the action of translations and the orthogonal group *O*(3) in , the latter one being comprised by the rotation group *SO*(3) and reflections, and regarded as a whole as the Euclidean group *E*(3). In this work, we have used the equivariant transformer (ET) in TorchMD-NET,^28^ which is an *SE*(3) -equivariant MPNN, i.e., equivariant to translations and rotations. Notice that ET does not incorporate parity equivariance and hence is not E(3)-equivariant like, e.g., NequIP.^29^ Like other EGNNs, it was primarily designed for the prediction of molecular energies and atomic forces to reconstruct potential energy surfaces of molecules and materials, respectively. By operating on a 3D point cloud given by atomic coordinates augmented with atomic numbers, it produces energy predictions , which are differentiated against input coordinates **r**. In that way, we obtain a conservative force field: . The equivariant transformer uses a distance filter implemented by (learnable) radial basis functions with a cosine cutoff encoding interatomic distances. Unlike PaiNN,^16^ ET uses a modified multihead attention mechanism that combines edge and node data and incorporates interatomic distances directly into the feature vectors. The output of ET comprises scalar and vector features. The scalar features are rotationally and translationally invariant, while the vector features have rotational equivariance. All internal operations maintain the equivariance of vector features, and therefore, equivariance is also preserved in the output. Scalar and vector features can be combined using a gated MLP,^15,16^ which also serves as an output head that can be modified depending on the task. Notice that the output head maps both scalar and vector features to a scalar value, and hence, the model output is invariant to translation and rotation.

For more details, we refer the interested reader to Thölke et al.^28^ For an overview of the equivariant transformer architecture, see Figure S1 of the Supporting Information (SI).

### State-of-the-Art Toxicity Predictive Models

We compare our results to current state-of-the-art ML models whenever benchmarks are accessible. AttentiveFP^23^ is a fully connected 2D graph neural network with an attention mechanism that works on nine precalculated atomic and four bond features to characterize atoms in the graph. Like AttentiveFP, D-MPNN^46^ is built on 2D graphs but uses directed message-passing encoding on precalculated atomic and bond features. AttrMask^24^ also uses a 2D graph but is pretrained by retrieving masked node and edge attributes. Lastly, GEM^4^ uses a geometry-based graph neural network architecture and several dedicated geometry-level self-supervised learning strategies to encode the molecular geometry. GEM introduces a so-called GeoGNN architecture that encodes the molecular geometries by modeling two separate graphs, the atom–bond and bond–angle graphs.^4^ The atom and bond representations are learned iteratively. But the network has two potential pitfalls. (1) Building two different graphs, especially building a bond-angle graph, is computationally expensive and might lack resolution.^16^ (2) GeoGNN does not use equivariant (higher-order) tensor features, which are important for molecular representation learning.^16,28,29^ For pretraining, the authors extract bond angles and distances from 3D conformers derived by RDKit using the classical Merck molecular force field (MMFF94). To the best of our knowledge, GEM is the current state-of-the-art on nearly all MoleculeNet^3^ data sets, and large-scale self-supervised pretraining has proven to be key. However, we emphasize two potential reasons for that. First, almost all publicly available toxicity data sets have a high-class imbalance. Second, the data sets often comprise just a few hundred to, at most, a few thousand samples. This means that models are expected to generalize on unseen data, though they may only have been trained on a few dozen positive labels. It is well-known that deep learning models struggle in the low data regime compared to classical machine learning techniques like Random Forests and, hence, large-scale pretraining seems to provide a potent helper. But this is beyond the scope of the present study, and we leave self-supervised learning as future work, i.e., our results can not be directly compared with methods like AttrMask^24^ or GEM^4^ that considerably rely on pretraining. It is worth noting that ET demonstrates performance comparable to that of these models. Nevertheless, we hypothesize that pretraining can potentially lead to superior results.

## Toxicity Data Sets

In this work, we utilize existing, and well-established data sources, namely, TDCommons,^38^ ToxBenchmark,^37^ and MoleculeNet.^3^ For the first two, we needed to generate the 3D conformers ourselves (see below), while for the MoleculeNet data sets, we used the 3D conformers provided by the GEOM^39^ data set. In GEOM, the authors used the CREST(40) software to generate conformations for various data sets and molecules from which we took Tox21, ToxCast, Sider, Clintox, BBBP, as well as BACE. All calculations were performed in a vacuum except for the BACE compounds, whose 3D conformers were obtained using GBSA implicit solvent model of water. The use of implicit water was considered to enhance the accuracy of conformational sampling for BACE compounds because solvent effects are known to dramatically change molecular properties as well as the outcome of reactions, leading to results that better reflect experimental observations.^47^ Notice that GEOM provides conformer rotamer ensembles (CREs), which we use in multiconformer training by selecting the most likely conformers sorted by their Boltzmann weights.

### Therapeutics Data Commons

Therapeutics Data Commons (TDCommons)^38^ has been set up recently to establish the first unifying platform to systematically access and evaluate ML across the entire range of therapeutics. TDCommons includes roughly 66 AI-ready data sets spread across 22 learning tasks, spanning the discovery and development of safe and effective medicines.^38^ TDCommons also provides an ecosystem of tools and community resources with the possibility of a systematic model evaluation attached to 29 public leaderboards. All resources are integrated and accessible via an open Python library.^38^

In the following, we briefly describe the five toxicity-related data sets from TDCommons that have been considered in this study: Ames, hERG, DILI, Skin Reaction, and LD50.

#### Ames

Mutagenicity means the ability of a drug to induce genetic alterations. Drugs that can cause damage to DNA can result in cell death or other severe adverse effects. Nowadays, the most widely used assay for testing the mutagenicity of compounds is the Ames experiment.^38^ The Ames data set provides 7,255 drugs with binary labels.

#### hERG

Human ether-à-go-go related gene (hERG) is crucial for the coordination of the heart’s beating. A drug blocking hERG could lead to severe adverse effects. Therefore, reliable prediction of hERG liability in the early stages of drug design is quite important to reduce the risk of cardiotoxicity-related attritions in the later development stages.^38^ The hERG data set provides 648 drugs with binary labels.

#### DILI

Drug-induced liver injury (DILI) is a fatal liver disease caused by drugs, and it has been the single most frequent cause of safety-related drug marketing withdrawals for the past 50 years.^38^ The DILI data set comes with 475 drugs and binary labels.

#### Skin Reaction

Repetitive exposure to a chemical agent can induce an immune reaction in inherently susceptible individuals that leads to skin sensitization.^38^ The Skin Reaction data set comes with 404 drugs and binary labels.

#### LD50

Acute toxicity LD50 measures the most conservative dose that can lead to lethal adverse effects for which 50% of a test population dies within a given time frame. The higher the dose, the more lethal the drug.^38^ The LD50 data set comes with 7,385 drugs and in contrast to all others is a regression data set.

### ToxBenchmark

Publicly available data sets to build and evaluate Ames mutagenicity prediction tools are very limited in terms of size and chemical space coverage. The goal of ToxBenchmark^37^ was to describe a new unique public Ames mutagenicity data set comprising about several thousand nonconfidential compounds together with their biological activity. This data set is similar to the Ames data set in TDCommons and contains 6,512 drugs with binary labels.

### 3D Conformer Generation

3D structure starting from SMILES strings is a challenging and computationally heavy procedure. Ab initio methods, such as DFT, are significantly more accurate than other force fields but also orders of magnitude more computationally demanding. As a balancing compromise, the CREST(40) code uses extensive sampling based on the much faster and yet reliable semiempirical extended tight-binding method (GFN2-xTB)^41^ to generate accurate 3D conformations. The semiempirical energies are much more accurate than classical force fields, accounting for electronic effects, rare functional groups, and bond-breaking/formation of labile bonds.^39,41^ Moreover, the search algorithm of CREST(40) is based on metadynamics (MTD), a well-established thermodynamic sampling approach that can efficiently explore the low-energy search space.^39,40^ Conformers are generated in an iterative manner of MTD and GFN2-xTB optimization, where those geometries are added to the CRE that overcome certain energy (12.0 kcal/mol) and RMSD (0.1 Å) thresholds with respect to the input structure. The procedure is restarted using the conformer as the input if a new conformer has a lower energy than the input structure. The three conformers of lowest energy then undergo two normal molecular dynamics (MD) simulations at 400 and 500 K, which are used to sample low-energy barrier crossings such as simple torsional motions. Finally, a genetic Z-matrix crossing algorithm is used and the results are added to the CRE. In the end, a normal-type convergence optimization separates the geometries into conformers, rotamers, and duplicates, where duplicates are deleted and conformers and rotamers added to the CRE.^39^ Geometry optimization and conformational search calculations were carried out considering the GBSA implicit solver model of water.

All data sets used in this work are listed in Table 1. We also report the number of retrieved compounds per data set as not every molecule could be optimized, so the data sets are not 100% complete. As can be seen in Table 1, the retrieval of 3D conformers for the SIDER data set is comparably bad, yielding only 95.1% of the original data. For all data sets, we deleted molecules for which we could not find any conformer.

**Table 1:** Overview and Statistics of All Data Sets Used in This Work Taken from MoleculeNet,^3^ TDCommons,^38^ and ToxBenchmark^37^^a^

Data set	Property	Tasks	Compounds	Recovered
Tox21	Qualitative toxicity	12	7,677	98.0%
ToxCast	Qualitative toxicity	617	8,405	98.0%
SIDER	Drug side effects	27	1,356	95.1%
ClinTox	Toxicity of failed approved drugs	2	1,438	98.7%
BACE	BACE-1 inhibition	1	1,511	99.9%
BBBP	Blood-brain barrier penetration	1	1,959	99.2%
Ames	Mutagenicity	1	7,269	99.8%
hERG	Coordination of heart beating	1	650	99.2%
DILI	Drug-induced liver injury	1	470	98.9%
Skin Reaction	Skin sensitization	1	403	99.7%
LD50	Acute toxicity	1	7,353	99.5%
ToxBenchmark	Mutagenicity	1	6,489	99.6%

a “Compounds” denotes the number of retrieved molecules per data set, and “Recovered” shows the percentage that could be recovered from the full data set.

### Data Set Splitting

For the MoleculeNet^3^ data sets, we use as train, validation, and test set splitting a scaffold split following the work done by Hu et al.^24^ which considers molecular chirality (https://github.com/snap-stanford/pretrain-gnns/blob/master/chem/splitters.py). Originally on MoleculeNet, only BBBP and BACE are reportedly trained on a scaffold split, but most recent work applies scaffold splitting on all data sets.^4^ For the TDCommons data sets, we use the provided Python API to retrieve a precalculated train, validation, and test scaffold split (https://tdcommons.ai/single_pred_tasks/tox/). For comparison, we have also obtained results performing random split and random scaffold split for all MoleculeNet data sets. The scaffold sets are shuffled and not deterministically ordered for random scaffold splitting. Hence, random scaffold splitting averaged over different seeds might give a better impression of generalization.

## Results and Discussion

We trained the equivariant transformer (ET)^28^ on the TDCommons,^38^ ToxBenchmark^37^ as well as the MoleculeNet^3^ data sets and used the area under the receiver operating characteristic curve (ROC-AUC) as an evaluation metric for all classification tasks. ET shows comparable performance to the state-of-the-art GEM^4^ on Tox21 and ToxCast, as well as notably better performance compared to (pretrained) 2D-graph-based models AttrMasking^24^ and AttentiveFP^23^ on four out of six data sets. For ToxBenchmark and TDCommons data sets, ET performs mostly on par with AttrMasking^24^ and AttentiveFP^23^ with significantly better performance on LD50. Surprisingly, ET trained on multiple conformers affects the performance of our baseline model trained on the most likely conformer. For single-conformer training, we also compared the baseline to randomly selected single conformers and did not find a significant change in performance. For example, we get a mean AUC of 0.831 ± 0.004 and 0.779 ± 0.01 by using a random conformer instead of the lowest in energy for Ames and Tox21 data sets. To have an idea of how different the conformers are from the lowest energy one, we have plotted the frequency plots of the energy differences and RMSDs in Figure S2 (Ames) and Figure S3 (Tox21) of the SI. For the model trained on LD50, the mean absolute error (MAE) was computed to evaluate the performance of ET since it is the only regression data set.

In our initial investigation, we conducted tests on various EGNNs including SchNet, PaiNN, and a self-designed body-ordered spherical harmonics-based EGNN. However, we did not observe substantial differences in performance among these models. The equivariant networks PaiNN and ET tested on par, while SchNet was a bit worse, as the network only learns invariant scalar features. Consequently, we made a decision to use the TorchMD-Net(ET) model, which is both well-established and computationally efficient, allowing us to investigate attention weights for enhanced explainability without compromising accuracy.

All TDCommons data sets, despite LD50, as well as ToxBenchmark, provide binary labels. For MoleculeNet, we deploy a multitask training for Tox21 (12 tasks), ToxCast (617 tasks), SIDER (27 tasks), and ClinTox (two tasks), and consequently, we report the average ROC-AUC across tasks. BACE and BBBP are also binary labeled.

The label distribution on all TDCommons data sets is balanced with almost 50/50 active and nonactive labels; hence, the ROC-AUC metric seems appropriate. However, the MoleculeNet data sets exhibit a significant imbalance in their label distribution. In such cases, using the Precision-Recall-AUC (PR-AUC) metric can provide more meaningful insights as ROC-AUC evaluations may be misleading. Indeed, the ROC curve can still suggest a good performance of the model even if most or all of the minority classes are misclassified, while the PR-AUC will indicate poor performance.^48^ Nonetheless, it is important to note that the PR-AUC depends on the baseline probability of positive labels, which can limit its expressiveness and usefulness for comparability across different data sets. Following recent works,^4,23,24^ we opt to evaluate the ROC-AUC instead of the PR-AUC to maintain comparability. If not stated differently, we selected the most likely conformer for every molecule, depending on the Boltzmann weight for single-conformer training. Whereas, for multiconformer training, we selected the three most likely conformers per molecule. Here, every conformer is given to the model as an independent instance. We also tested five and ten conformers and found that the selection of three conformers worked best on the test set, while for training, the higher the number of conformers, the faster the convergence. We further tested two different settings. First, following ref (49), we employ multi-instance learning, where every bag contains the molecules conformers. Here we could not find any improvements. In the second approach, we randomly select a conformer before every batch step with a probability of 50%, and otherwise, the lowest energy conformer is used. This significantly boosted the performance on Tox21, but not on the other data sets. Notice that, as a proof-of-concept study, we assume here for simplicity that the most likely conformation is also the most relevant for toxicity prediction. To ensure consistency and comparability with existing research, these conformations are used for testing and validation of the models, leaving the respective data distribution unperturbed from multiconformer augmentation. We perform five different seed runs for all data sets and report the mean AUC with its standard deviation.

We further introduce a transformer model, SMILES-T, that works based on Simplified Molecular Input Line Entry System (SMILES) strings^50^ and directly compares to our purely geometry-based ET model. For the SMILES-based transformer, we use tokenized and integer-transformed representations as input similar to Schwaller et al.^51^

Moreover, in separate studies, we add the total energy of the molecules calculated with GFN2-xTB^41^ as additional node features besides atom types to enrich the model with a physical prior. We also tested adding the total energy as a molecular feature after node aggregation and before the output head, but we found no difference in performance. To the best of our knowledge, there is a lack of theoretical and experimental research investigating the potential correlation between a drug’s toxicity and its (intensive/extensive) physicochemical properties such as the total energy. Thus, our goal is to address this notoriously difficult task, which has yet to be fully understood, by machine learning approaches; these could supply valuable insights into toxicity. Accordingly, we initialize every node in the graph with the respective atom types and the energies of the molecular system.

The transformer architecture further allows us to investigate the learned importance of atomic pairs and substructures, potentially in correlation with certain physicochemical properties. We here restrict ourselves to an attention analysis and leave a deeper, more thorough explainability investigation for later studies.

More training details and a list of important hyperparameters can be found in Table S1 of the SI.

### Benchmark: TDCommons and ToxBenchmark

The overall performance of ET and the comparison to other methods are summarized in Table 2. ET (single) and ET (multi) denote single- and multiconformer training, respectively. For the sake of clarity, we compress the results for Random Forest,^52^ XGBoost,^53^ BaseBoosting^54^ and Support Vector Machines (SVMs)^55^ as “Fingerprints-based” method because they are based on different kinds of descriptors like circular extended-connectivity fingerprints (ECFPs).^56^ We observe that the methods relying on precalculated descriptors (fingerprints) outperform all other methods on four out of five data sets. ET is mainly performing on par with AttrMasking^24^ and AttentiveFP^23^ but performs better on LD50. On the Skin Reaction data set, we could not find any other benchmarks. To the best of our knowledge, the Ames mutagenicity provided by ToxBenchmark^37^ has been evaluated only once with an SVM using a random 5-fold cross-validation scheme.^5^ We compare this result with the performance of ET on five random splits and achieve a significantly better performance. For completeness, the AUC result on a scaffold split, as described above, is 0.848 ± 0.004. The SMILES-Transformer performs significantly poorly compared to all other methods, partly by a wide margin, suggesting that SMILES-based structure–activity relationship modeling without pretraining lacks expressiveness compared to the descriptor and 2D/3D graph-based models, respectively.

**Table 2:** Overall Performance for Classification and Regression Tasks on Toxicity-Related Data Sets of TDCommons^38^ and ToxBenchmark^37^^a^

**Data set**	**Ames** ↑	**hERG** ↑	**DILI** ↑	**Skin Reaction** ↑	**LD50** ↓	**Tox- Benchmark** ↑
**No. molecules**	7,269	650	470	403	7,353	6,489
**Label dist.**	0.55:0.45	0.68:0.32	0.5:0.5	0.68:0.32	2.54/0.95	0.53:0.47
AttrMasking	0.842 ± 0.008	0.778 ± 0.046	0.919 ± 0.008	-	0.685 ± 0.025	-
AttentiveFP	0.814 ± 0.008	0.825 ± 0.007	0.886 ± 0.015	-	0.678 ± 0.012	-
Fingerprint-based	**0.865** ± 0.002	**0.875** ± 0.003	**0.937** ± 0.004	-	**0.588** ± 0.005	0.86 ± 0.01
SMILES-T	0.697 ± 0.011	0.703 ± 0.056	0.760 ± 0.041	0.633 ± 0.051	0.715 ± 0.012	0.720 ± 0.014
ET (single)	0.836 ± 0.003	0.839 ± 0.017	0.878 ± 0.013	0.662 ± 0.033	0.653 ± 0.008	**0.881** ± 0.008
ET (multi)	0.804 ± 0.004	0.763 ± 0.021	0.885 ± 0.030	0.581 ± 0.055	0.660 ± 0.01	**0.879** ± 0.005

a For Ames, hERG, DILI, Skin Reaction, and ToxBenchmark, we report the normalized label distribution as active:inactive. For LD50, the mean and standard deviation is given. The equivariant transformer is denoted as ET. Here, (single) and (multi) differentiate between single- and multi-conformer training. We report the standard deviation for five different seed runs in subscripts. Two numbers are written in bold in one column if standard deviations overlap.

Our results also suggest that multiconformer training does not help the model generalize better than single-conformer training across all the data sets and even decreases the performance significantly on hERG and Skin Reaction. We hypothesize that including conformers increases overfitting on the training data distribution only rather than improving generalization performance on the test set. Especially, when dealing with scaffold split data, adopting a multiconformer training approach may not provide further value for generalization on unseen scaffolds.

**Figure 2:**
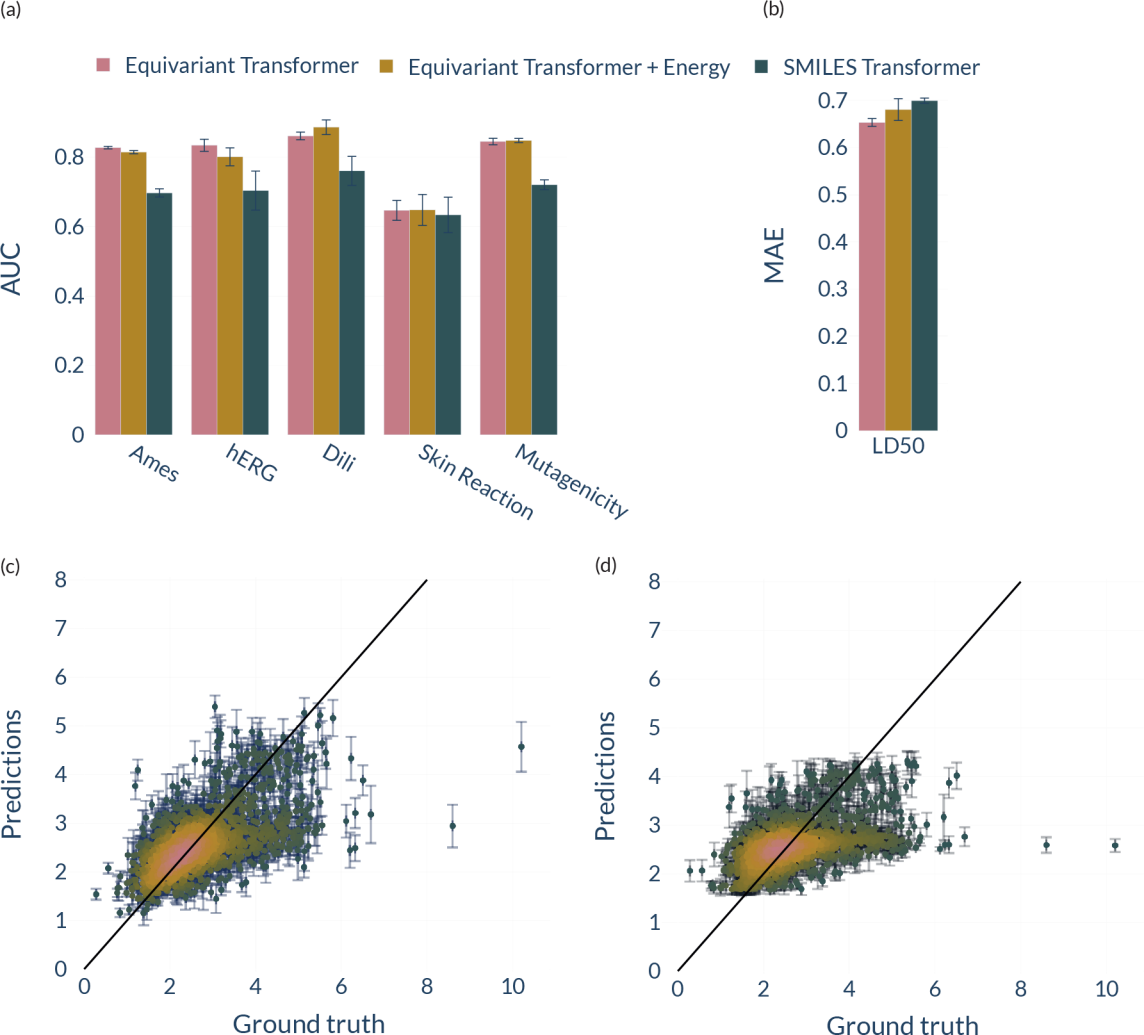
(a, b) Performance of ET, ET augmented with energies, and a SMILES-based transformer on the TDCommons and ToxBenchmark data sets. We report the standard deviation for five different seed runs as error bars. The metric of LD50 (b) is the mean absolute error (MAE), so lower is better. Otherwise, higher is better. (c, d) ET (c) and SMILES-Transformer (d) performance on LD50 compared to the ground truth labels. The brighter the color, the more predicted points. We report the standard deviation for five different seed runs as error bars.

In Figure 2a, we compare ET normally trained, ET trained with additional energy features, and a SMILES-based transformer. It can be seen that additional information about the energy of the molecules does not help the model in generalization but introduces a higher deviation between the results for different splits. Also, the convergence time of the model remained almost unaltered while training with the extra feature. As a further test, we investigated the model’s performance when trained in a multitask setting predicting energies and respective toxicities. Still, we again found that this significantly hurts the model’s performance. Hence, we conclude that there does not seem to be a correlation between the energetics of a molecule and its toxicity activity. One reason for the inferior performance might be that toxicity is often a complex, multifactorial process that might also involve a drug’s metabolic behavior. Moreover, from a chemical perspective, a molecule can be classified as toxic based on its chemical composition, which may contain hydrophobic atoms, electron-donating groups, or electron-withdrawing groups, making toxicity an intensive property. However, the total energy of a molecule is predominantly determined by its molecular size rather than the functional groups that are present within its structure. Figure 2c and d display the correlation plot between the predictions made by ET and SMILES-T, respectively, trained on LD50 compared to the ground truth labels. One can see that ET is showing good performance on most molecules but also clearly fails on a few dozen molecules, similar to SMILES-T. Nevertheless, as can be seen from the density encoding (the brighter, the more points), ET’s predictions mostly follow the 45-degree line, whereas SMILES-T diverges.

### Benchmark: MoleculeNet

**Figure 3:**
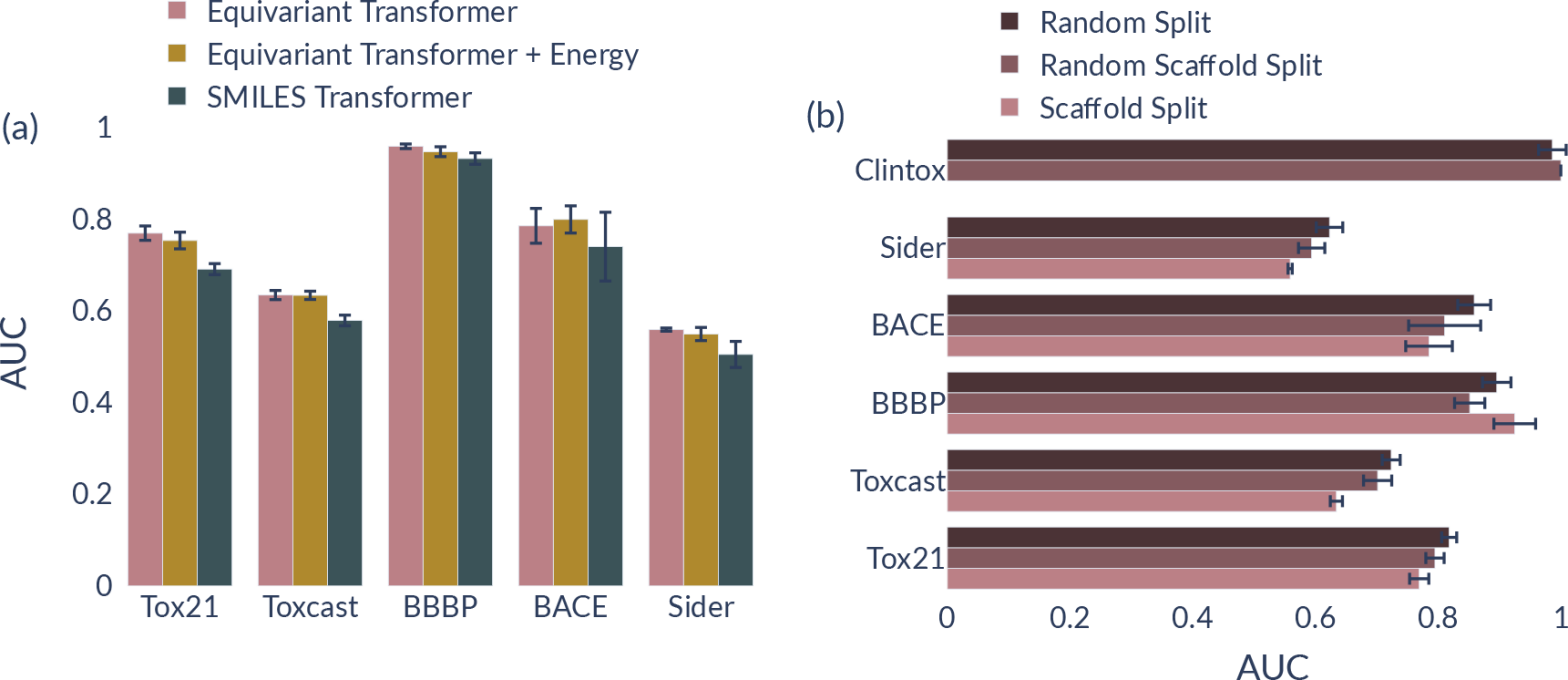
(a) Performance of ET, ET augmented with energies, and a SMILES-based transformer on the MoleculeNet data sets. We report the standard deviation for five different seed runs as error bars. (b) Performance of ET on different data set splitting methods. We report the standard deviation for five different seed runs as error bars.

The overall performance of ET on the MoleculeNet data sets and the comparison to other methods is summarized in Table 3. We observe that GEM^4^ is significantly outperforming all other methods on three out of six tasks and is on par with D-MPNN^46^ on ClinTox. GEM uses a large-scale pretraining scheme that uses information about the geometry of the molecules by taking into account distances and a separate bond-angle graph. In that regard, the results obtained by these methods are not directly comparable to ours, as mentioned before. Nevertheless, ET is outperforming GEM in the multiconformer setting with random conformer augmentation on Tox21 and works on par on ToxCast and additionally better than AttrMasking^24^ and D-MPNN^46^ on BACE. For all tasks and, similar to TDCommons and ToxBenchmark data sets, there is not a significant difference between single- and multiconformer training. However, on SIDER, the failure of ET could be related to the fact that the data are only retrieved to 95.1%. The training on ClinTox has been proven to be highly volatile, as the standard error shows. Lastly, the performance of ET and SMILES-T on BBBP is considerably better compared with the other methods. The results were carefully examined, and no overlaps or irregularities were found during the training and evaluation of the predictive models. Nevertheless, we cannot exclude an error in the data. Notice that ET significantly outperforms the SMILES-based transformer across all data sets (see Table 3 and Figure 3a), as it also happened for the data sets of TDCommons^38^ and ToxBenchmark.^37^ In the same breath, we have found that energy augmentation does not improve the ET model, another example that energetics does not correlate with toxicity activity. Figure 3b summarizes the performance of ET trained on three different data splits, random splitting, random scaffold splitting, and scaffold splitting. The scaffold splits are carried out considering chirality as described above. We can see that the data set split has a significant impact on the performance of the model. As expected, we get the best performances for random splitting directly, followed by random scaffold splitting.

**Table 3:** Overall Performance for Classification Tasks on Toxicity-Related Datasets of MoleculeNet^3^^a^

**Data set** ↑	**Tox21**	**ToxCast**	**SIDER**	**ClinTox**	**BACE**	**BBBP**
**No. molecules**	7,677	8,405	1,356	1,438	1,511	1,959
**Label dist.**	0.06:0.77:0.17	0.03:0.27:0.70	0.57:0.43:-	0.51:0.49:-	0.54:0.46:-	0.76:0.24:-
**No. tasks**	12	617	27	2	1	1
D-MPNN	0.759 ± 0.007	0.655 ± 0.003	0.57 ± 0.007	**0.906** ± 0.006	0.809 ± 0.006	0.724 ± 0.004
AttentiveFP	0.761 ± 0.005	0.637 ± 0.002	0.606 ± 0.032	0.847 ± 0.003	0.784 ± 0.022	0.643 ± 0.018
GEM	0.781 ± 0.001	**0.692** ± 0.004	**0.672** ± 0.004	0.901 ± 0.013	**0.856** ± 0.011	0.724 ± 0.004
SMILES-T	0.691 ± 0.011	0.578 ± 0.011	0.504 ± 0.028	0.819 ± 0.045	0.739 ± 0.075	0.931 ± 0.012
ET (single)	0.780 ± 0.004	**0.685** ± 0.009	0.606 ± 0.01	0.851 ± 0.027	0.832 ± 0.009	**0.960** ± 0.03
ET (multi)	**0.789** ± 0.003	0.623 ± 0.008	0.560 ± 0.011	0.843 ± 0.012	0.816 ± 0.013	**0.955** ± 0.008

a The normalized label distribution is denoted as active:inactive:nan. The equivariant transformer is denoted as ET. Here, (single) and (multi) differentiate between single- and multi-conformer training. We report the standard deviations for five different seed runs as subscripts. Two numbers are written in bold in one column if the standard deviations overlap.

**Figure 4:**
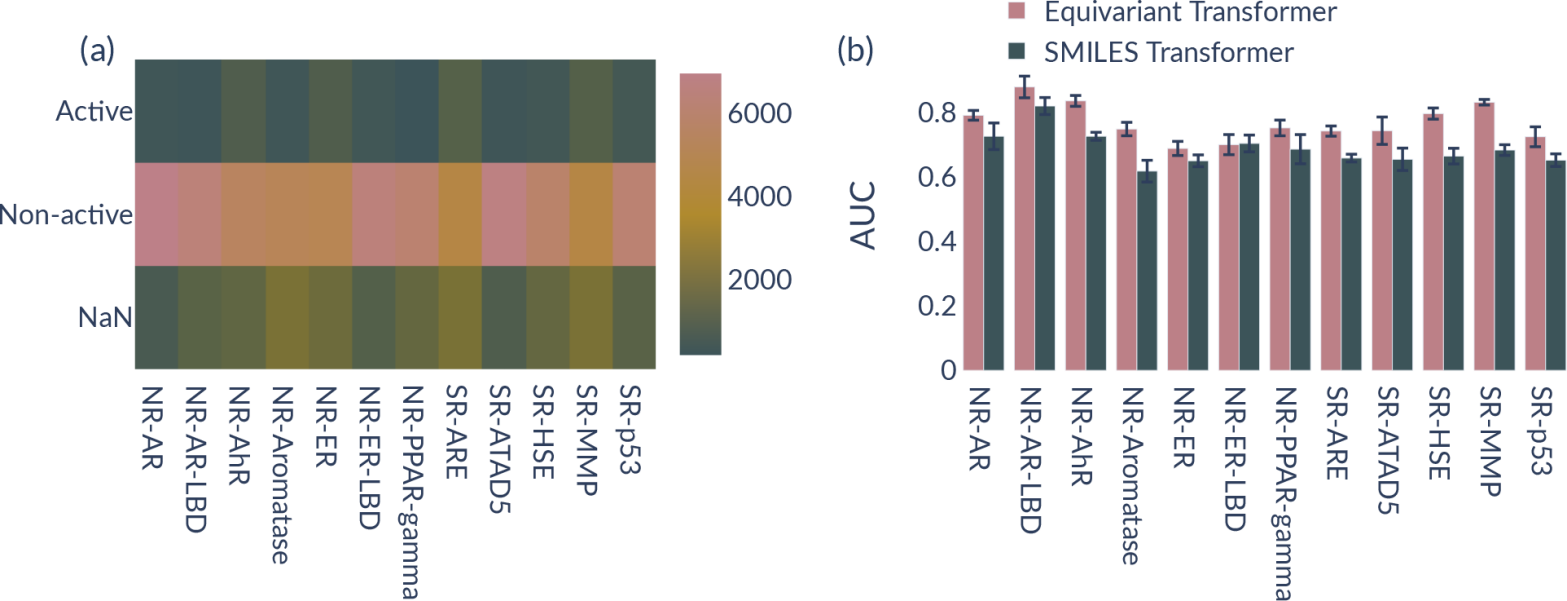
(a) Label distribution on the Tox21 data set for all 12 tasks. (b) Performance of ET compared to a SMILES-based transformer for all 12 tasks of Tox21 (scaffold split). We report the standard deviation for five different seed runs as error bars.

Then we investigate the performance of ET and SMILES-T on the 12 different tasks of the Tox21 data set; see Figure 4. Figure 4a presents the label distribution for these tasks, while Figure 4b shows the performance of ET and SMILES-T per task. Here, NR is the abbreviation for nuclear receptor signaling pathways and SR stands for stress response pathways. Nuclear receptors are crucial in cell communication and control and play an important part in development, metabolism, and proliferation and, hence, in toxicology, in general.^57^ The NR tasks are divided into estrogen (NR-ER/NR-ER-LBD) and androgen receptors (NR-AR/NR-AR-LBD), respectively, to study the endocrine system and further into antagonists of the aromatase enzyme (NR-Aromatase), the aryl hydrocarbon receptor (NR-AhR) as well as peroxisome proliferator-activated receptors (NR-PPAR-gamma).^57^ The stress response (SR) tasks are divided into the antioxidant response element signaling pathway (SR-ARE), heat shock factor response element (SR-HSE), DNA damage (ATAD5), mitochondrial membrane potential (SR-MMP), and finally, the p53 pathway (SR-p53).^57^ As the number of active, nonactive, and nonavailable samples is mostly the same across tasks, we do not expect a correlation between label distribution and performance. In fact, one can see that both models show a clear pattern with a large divergence depending on the task. Interestingly, both models perform on average best for NR-AR-LBD and comparably poorly for NR-ER and NR-ER-LBD. Using 3D geometries seems to help the most compared to SMILES strings on all SR-related tasks, especially for SR-MMP and SR-HSE. However, ET performs just insignificantly better for NR-ER and NR-ER-LBD compared to the SMILES-based transformer.

### Geometry Dependency

To verify whether geometry information is crucial for toxicity prediction, we here ablate the distance input for ET on Ames, LD50, and Tox21 by training on only chemical elements (Table 4). In other words, the graph consists of only node features given by atom types. We further test two different cutoffs, 2 and 12 Å, to evaluate covalent and long-range dependencies, respectively. Interestingly, in contrast to LD50 and Tox21, for Ames geometry information helps statistically (0.836 ± 0.003) but is not most crucial for the performance. A larger cutoff significantly hurts generalization significantly. Hence, Ames toxicity seemingly can be relatively well predicted only by considering covalent interactions and chemical elements. For LD50 (0.653 ± 0.008) and Tox21 (0.780 ± 0.004), we can see that geometry information is crucial. Especially on LD50, long-range information is also of significant importance.

**Table 4:** Ablation Study on Ames, LD50, and Tox21 Evaluated without Geometry-Aware Training^a^

**Data set**	**Ames** ↑	**LD50** ↓	**Tox21** ↑
cutoff 2 Å	**0.797** ± 0.008	0.928 ± 0.019	0.692 ± 0.008
cutoff 10 Å	0.668 ± 0.009	**0.702** ± 0.013	-
cutoff 12 Å	-	-	**0.713** ± 0.009

a Here, we exclude the geometrical input, so the model sees nodes comprising only the chemical composition. We also test the covalent and long-range dependence by comparing two different cutoffs. A cutoff of 2 Å covers covalent interactions only, whereby a cutoff of 10 or 12 Å covers non-local interactions. We report the standard deviation for five seed runs as subscripts.

### Explainability: Attention Weights Analysis

We provide an attention weights visualization for three different data sets: Ames, LD50, and Tox21; see Figure 6. Inference on two selected molecules taken from the test set was run, and then the respective attention matrix from all attention heads in all layers was saved. We randomly selected molecules for inference for the Ames data set until we got two toxic ones. In the molecular selection for the LD50 data set, we have considered only molecules with LD50 values lower than 2.0, whereby the mean value of the data set is 2.5. On this subset, the randomly selected molecules have LD50 values of 1.77 and 1.94, respectively. However, for Tox21, for the molecular selection, we randomly sampled from a subset of all molecules that are labeled as active on both SR-MMP and SR-HSE tasks. Following the work done by Thölke et al.,^28^ who used Attention rollout^58^ under the single head assumption, one can get a single attention matrix per sample. In doing so, the attention scores of atom pairs throughout the molecule are computed. The dotted product of each attention map in every layer is evaluated progressively. The obtained attention weights highlight the importance that the model gives to atomic pairs in a message-passing architecture.

**Figure 5:**
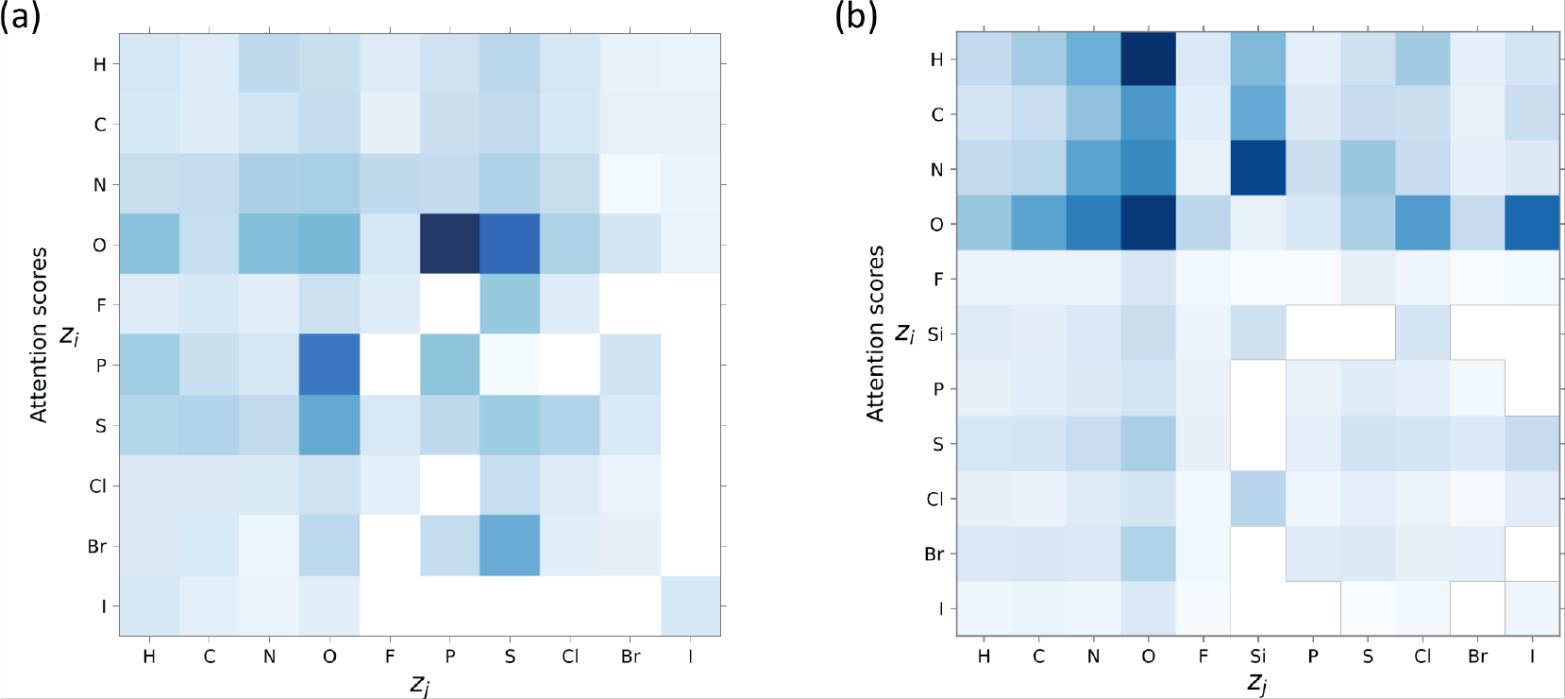
Heat maps of attention scores on the (a) Ames and (b) LD50 test sets. The darker the color, the more attention the model gives on average to atom *Z*_*i*_ attending to atom *Z*_*j*_. Here, the attention scores are calculated based on attention rollout^58^ and, in the end, summed up for every atom type.

We first computed the attention score heat map for Ames and LD50 test sets (Figure 5a,b). Subsequently, we compared these scores with the corresponding bond probabilities obtained from the respective data sets (see Figures S4 and S5 of the SI). One can see that for both data sets, the model does not follow the data statistics (by simply learning the bond probabilities) but highlights specific pairwise interactions within the molecules. For the Ames data set, the highest attention scores were observed in interactions involving O atoms paired with P and S atoms. In contrast, for the LD50 data set, these scores were primarily associated with interactions between O atoms and certain halogen atoms, as well as N–Si, H–O, and O–O pairs. It is worth mentioning that the LD50 model identified a considerably larger number of important interactions compared to the Ames model, which coincides with the evaluation of covalent vs long-range dependencies, whereby in contrast to Ames, LD50 predictions are significantly positively influenced by long-range coverage. The results obtained for the Tox21 test set are plotted in Figures S7 and S8 of the SI.

**Figure 6:**
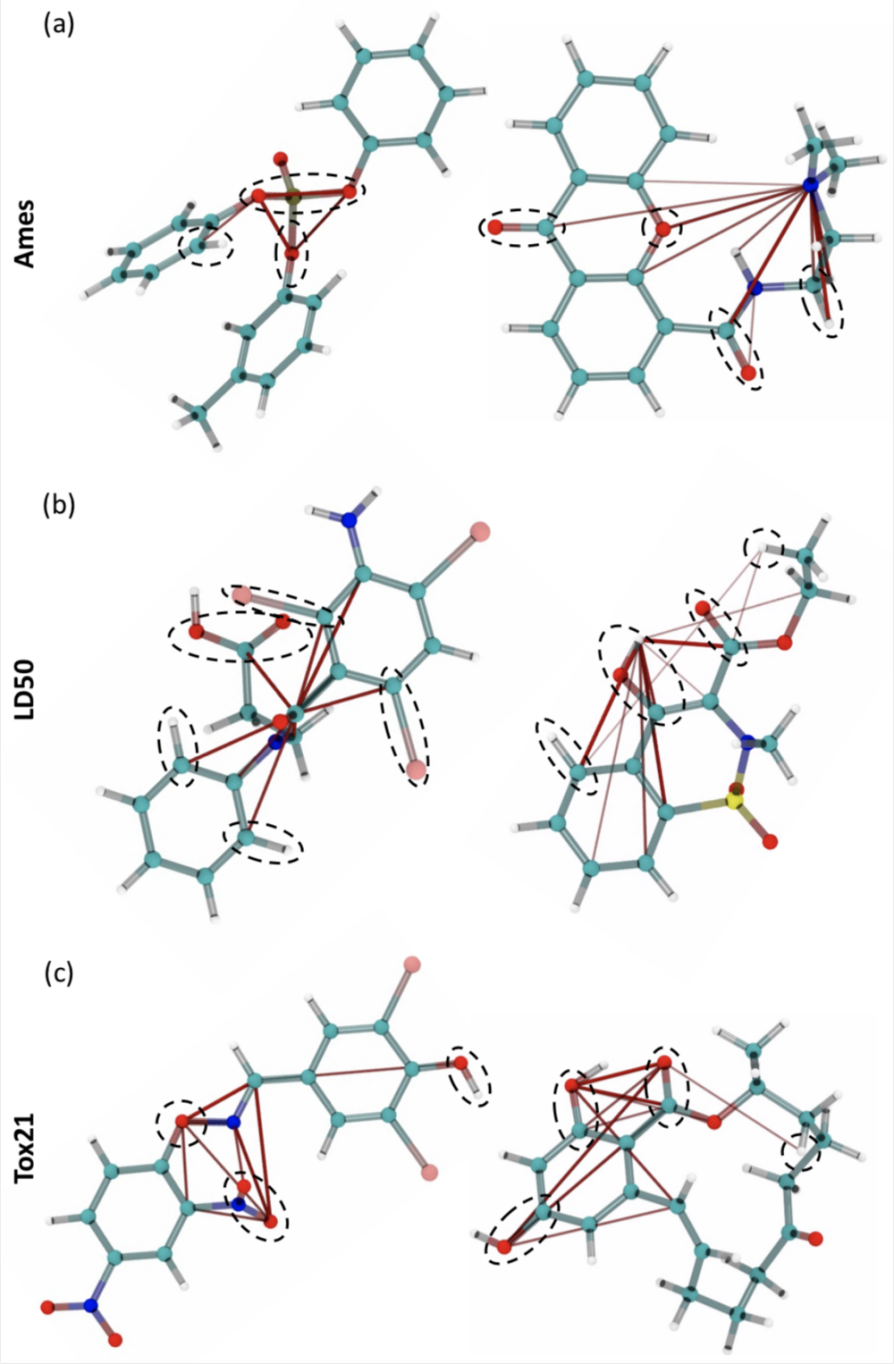
Visualization of attention weights using Attention rollout^58^ on (a) Ames, (b) LD50, and (c) Tox21 test sets. The thickness of the attention lines encodes the amplitude of attention. The dashed circles highlight chemically important functional groups that are also recognized by the network: hydrophobic atoms, electron-donating groups, electron-withdrawing groups, and polar groups.

In order to develop a more profound comprehension of these heat maps, we focused our study on the ten highest attention weights for specific molecules, which are represented in the images as red (negative) or blue (positive) strings. The thickness of the strings reflects the strength of the absolute weight. Our findings align with established chemical principles that describe the toxicity activity within a molecule.^59^ For instance, in addition to emphasizing hydrophobic (C and H) atoms, the highest attention weights in Ames molecules encompass electron-donating and electron-withdrawing groups, such as SP3 oxygen and functional groups with carbonyl, respectively (Figure 6a). Furthermore, our analysis brings attention to organophosphorus compounds, which are known to inhibit certain enzymes. In the same breath, Figure 6b shows LD50 molecules where the attention weights primarily establish connections between hydrophobic atoms, carbonyl/hydroxyl/carboxyl groups, and atoms interacting with Iodine (halogen). Besides the aforementioned chemical fragments, the attention map for Tox21 molecules highlights the presence of a nitro group known for its exceptional electron-withdrawing properties; see Figure 6c. Intriguingly, we have discovered that certain molecules exhibit a highly localized attention weight map, whereas others showcase widespread coverage across the molecular structure. This could be an indication of the existence of distinguishable toxicity centers; however, additional investigation is needed to ascertain their significance and implications.

**Figure 7:**
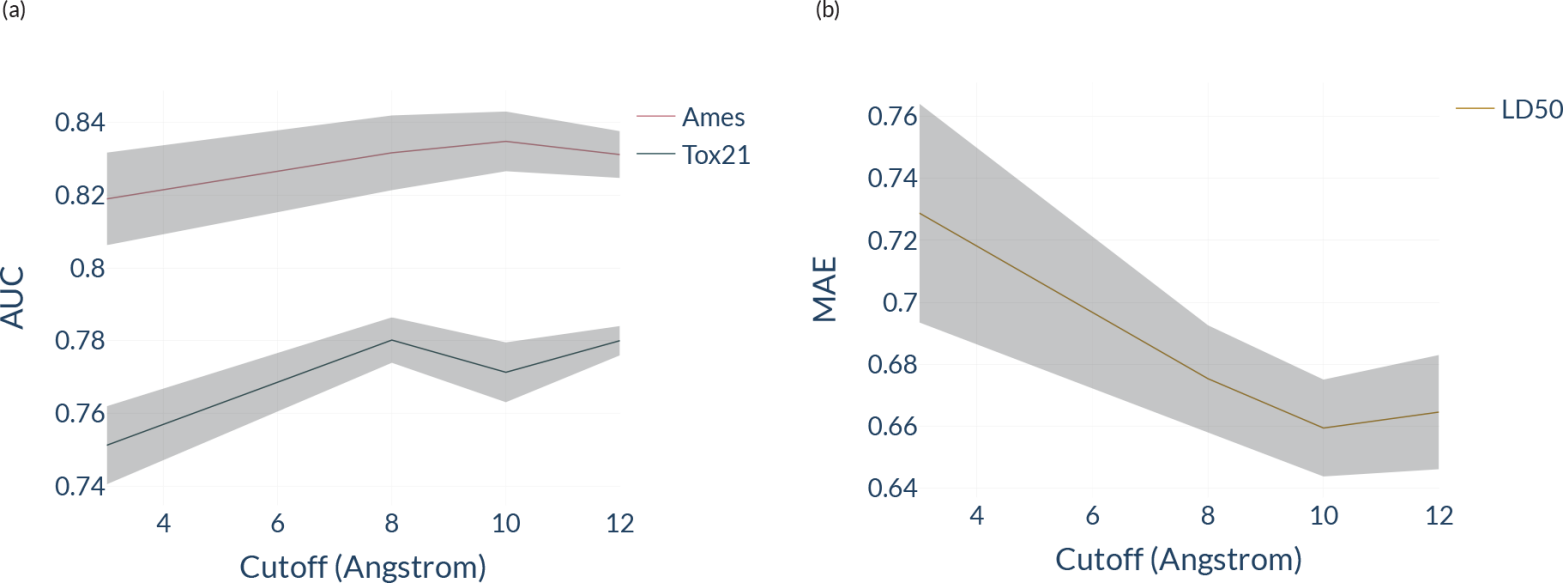
Predictive performance of ET on (a) Ames, Tox21, and (b) LD50 as a function of the cutoff distance used in the message-passing layers. We report the standard deviation for five seed runs with a shaded area.

To further investigate this, we modify the cutoff in ET used to train the ML models for these three data sets (see Figure 7). As expected from the previous section, we also found that the geometry-aware model is strongly influenced by the cutoff for LD50 and Tox21 compared to Ames. This is clear evidence that long-range effects may significantly influence the prediction performance for certain toxicity data sets, which is expected for the dimensions of these molecules and their large chemical/structural complexity. Notice that similar cutoff dependence has also been observed in ML force fields for calculating quantum-mechanical properties of organic molecules and materials, i.e., the larger cutoff, the more accurate the ML force field.^26,31^ In brief, these exciting findings have shown essential features of using EGNNs to develop explainable and reliable ML models for toxicity prediction.

## Conclusion

Molecular property prediction and QSAR modeling are crucial to diverse drug discovery pipelines. However, finding efficient to calculate and yet highly reliable molecular representations is challenging and, consequently, heavily researched. Particularly, current representations do not cover (well) all geometric symmetries (e.g., invariant to translation and rotations, while using equivariant tensor features internally) of a molecule, which might be crucial for correctly predicting molecular properties and ADMET end points. To provide a step toward 3D modeling in toxicity prediction, in this work, we investigated and benchmarked an equivariant graph transformer model (ET)^28^ that only considers the geometry of a conformer and its atom types on several toxicity-related data sets. In doing so, we used precalculated 3D conformers^39^ from the well-established MoleculeNet^3^ data sets and calculated high-quality conformers for five toxicity data sets provided by TDCommons,^38^ and ToxBenchmark^37^ on our own. We showed that ET produces comparable results to state-of-the-art 2D graph-based models and outperforms a SMILES strings-based transformer model across all data sets and tasks. For all TDCommons data sets, fingerprint-based models perform better than all other methods, including ET. We expect that this may change when (toxicity) data sets further scale up in size, such that deep learning models have more samples to learn from than just a few hundred up to a few thousand compounds. Our results also suggest that the prediction performance of ET is practically independent of the conformer selection or the number of conformers per molecule used during the training process. Nevertheless, we showed that geometry input, in general, is crucial for the performance across data sets, although with varying significance. Moreover, we studied the role of an additional quantum-mechanical feature, such as the total energy, in predicting the toxicity activity of molecules. We found that ET’s performance does not improve, which reveals a lack of correlation between energetics and toxicity activity. This result challenges the idea of a purely quantum-mechanical description of molecules for developing toxicity prediction models; further investigation is necessary for this subject. It has also been demonstrated that information about the 3D geometry is beneficial for all tasks in the Tox21 data set related to stress response pathways, outperforming the SMILES-based model by a considerably larger margin than on other tasks. Lastly, information about the reasoning of ET via attention weight analysis gives interesting and chemically meaningful insights into the mechanics of the model that might be helpful for researchers and practitioners. Hence, our findings provide valuable insights into developing reliable toxicity predictive models using the 3D representation of molecules in an ET framework.

## Data Availability

The conformer data sets and trained toxicity models will be published upon acceptance of this work. The code has been made available at https://github.com/jule-c/ET-Tox, and the processed data as well as pretrained models for training and testing can be downloaded from https://zenodo.org/record/7942946. We can provide the full list of conformers as XYZ files upon request.

## Supporting Information Available

Further details on equivariant transformer’s architecture, computational settings, and hyperparameters that are used for training the models; data statistics, attention analysis, and more training results (PDF)
