# Supplementary material for: Equivariant Graph Neural Networks for Toxicity Prediction
Source: Chem Res Toxicol. 2023 Sep 10;36(10):1561–73. doi: 10.1021/acs.chemrestox.3c00032 (PMC10583285; doi:10.1021/acs.chemrestox.3c00032)
Supplement: Supplementary file 1 — tx3c00032_si_001.pdf [file tx3c00032_si_001.pdf]

Supporting Information (SI) for:

Equivariant Graph Neural Networks for Toxicity Prediction

Julian Cremer,[*a,b] **Leonardo Medrano Sandonas,**[*c] **Alexandre Tkatchenko**[c]
Djork-Arné Clevert,[b] **Gianni De Fabritiis,**[a,d]

[a] *Computational Science Laboratory, Universitat Pompeu Fabra, Barcelona Biomedical Research Park (PRBB), Carrer Dr. Aiguader 88, 08003, Barcelona, Spain*
[b] *Machine Learning Research, Pfizer Worldwide Research Development and Medical, Linkstr.10, Berlin, Germany*
[c] *Department of Physics and Materials Science, University of Luxembourg, L-1511 Luxembourg City, Luxembourg*
[d] *ICREA, Passeig Lluis Companys 23, 08010 Barcelona, Spain*

[*] Corresponding authors: Julian Cremer (julian.cremer@upf.edu),
Leonardo Medrano Sandonas (leonardo.medrano@uni.lu)

1 Equivariant Transformer

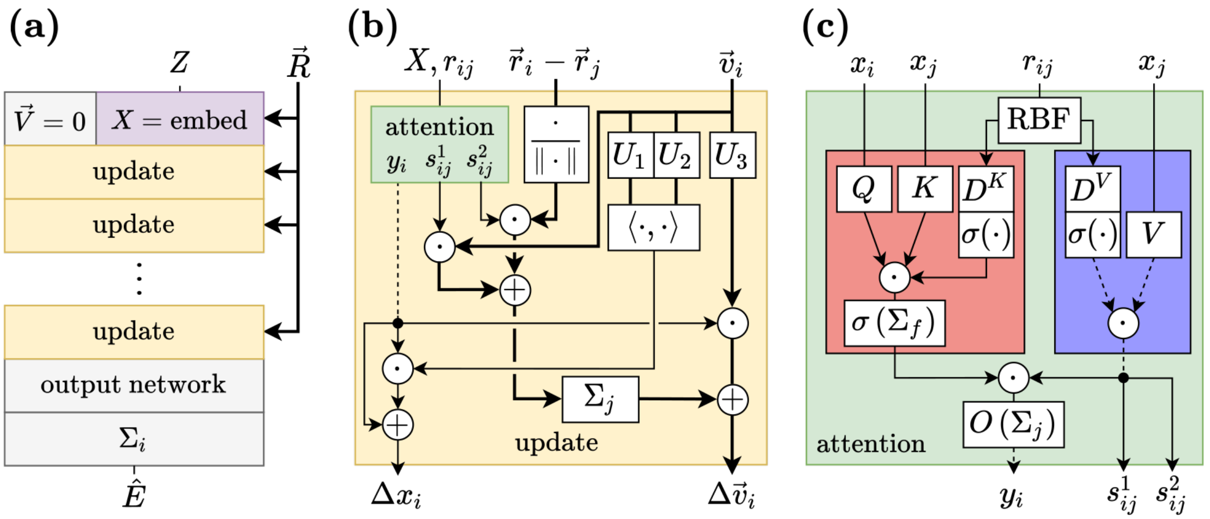

Figure S1 Overview of the equivariant transformer architecture. Thin lines: scalar features in $\mathbf{R}^F$, thick lines: vector features in $\mathbf{R}^{3F}$, dashed lines: multiple feature vectors. (a) The transformer consists of an embedding layer, several update layers, and an output network. (b) Residual update layer including attention-based interatomic interactions and information exchange between scalar and vector features. (c) Modified dot-product attention mechanism, scaling values (blue) by the attention weights (red)

2 Training Details

Table S1 reports the main hyperparameters used to train the equivariant transformer. Across datasets, we used mostly the same hyperparameters and only changed the batch size and the cutoff. We trained all models using early stopping with a patience of 25 epochs. For the reduce-on-plateau scheduler, we used a learning rate factor of 0.75 and a learning rate patience of 5. No warmup steps have been used.

Table S1 Hyperparameters for training the equivariant transformer model.

optimizer	AdamW
scheduler	reduce on plateau
learning rate	1e-4
weight decay	1e-5
batch size	8, 16
activation	silu
attention activation	silu
layernorm on vectors features	whitened
cutoff	8.0, 10.0, 12.0
no. of attention heads	8
no. of layers	5
embedding dimension	128
no. of RBFs	64

3 Training Results

Table S2 Overall performance for classification and regression tasks on toxicity-related datasets of TDCommons and ToxBenchmark. LD50 is a regression dataset, and the performance measure is RMSE. All other datasets are classification tasks and evaluated with PR-AUC. We also report a Null-Model, which outputs the label occurring most often in the dataset and, for LD50, the mean value. For Ames, hERG, DILI, Skin Reaction, and ToxBenchmark we report the normalized label distribution as active:inactive. For LD50, the mean and standard deviation is given. The equivariant transformer is denoted as ET. Here, (single) means single-conformer training.

**Dataset**	**Ames** ↑	**hERG** ↑	**DILI** ↑	**Skin Reaction** ↑	**LD50** ↓	**Tox-Benchmark** ↑
**No. molecules**	7,269	650	470	403	7,353	6,489
**Label dist.**	0.55:0.45	0.68:0.32	0.5:0.5	0.68:0.32	2.54/0.95	0.53:0.47
ET (single)	0.867	0.845	0.88	0.66	0.89	0.93
Null-Model	0.5	0.5	0.5	0.5	1.10	0.5

Table S 3 Overall performance for classification tasks on toxicity-related datasets of MoleculeNet. The normalized label distribution is denoted as active:inactive:nan. The metric used is PR-AUC. The equivariant transformer is denoted as ET. Here, (single) means single-conformer training. The Null-Model outputs the most often occurring label (e.g., for Tox21, the Null-Model only outputs 0 [inactive]) and is evaluated on both ROC- as well as PR-AUC.

**Dataset** ↑	**Tox21**	**ToxCast**	**SIDER**	**ClinTox**	**BACE**	**BBBP**
**No. molecules**	7,677	8,405	1,356	1,438	1,511	1,959
**Label dist.**	0.06:0.77:0.17	0.03:0.27:0.70	0.57:0.43:-	0.51:0.49:-	0.54:0.46:-	0.76:0.24:-
**No. tasks**	12	617	27	2	1	1
ET (single)	0.62	0.6	0.57	0.57	0.84	0.97
Null-Model (ROC-AUC)	0.5	0.5	0.5	0.5	0.5	0.5
Null-Model (PR-AUC)	0.10	0.22	0.47	0.5	0.45	0.78

4 Dataset Statistics

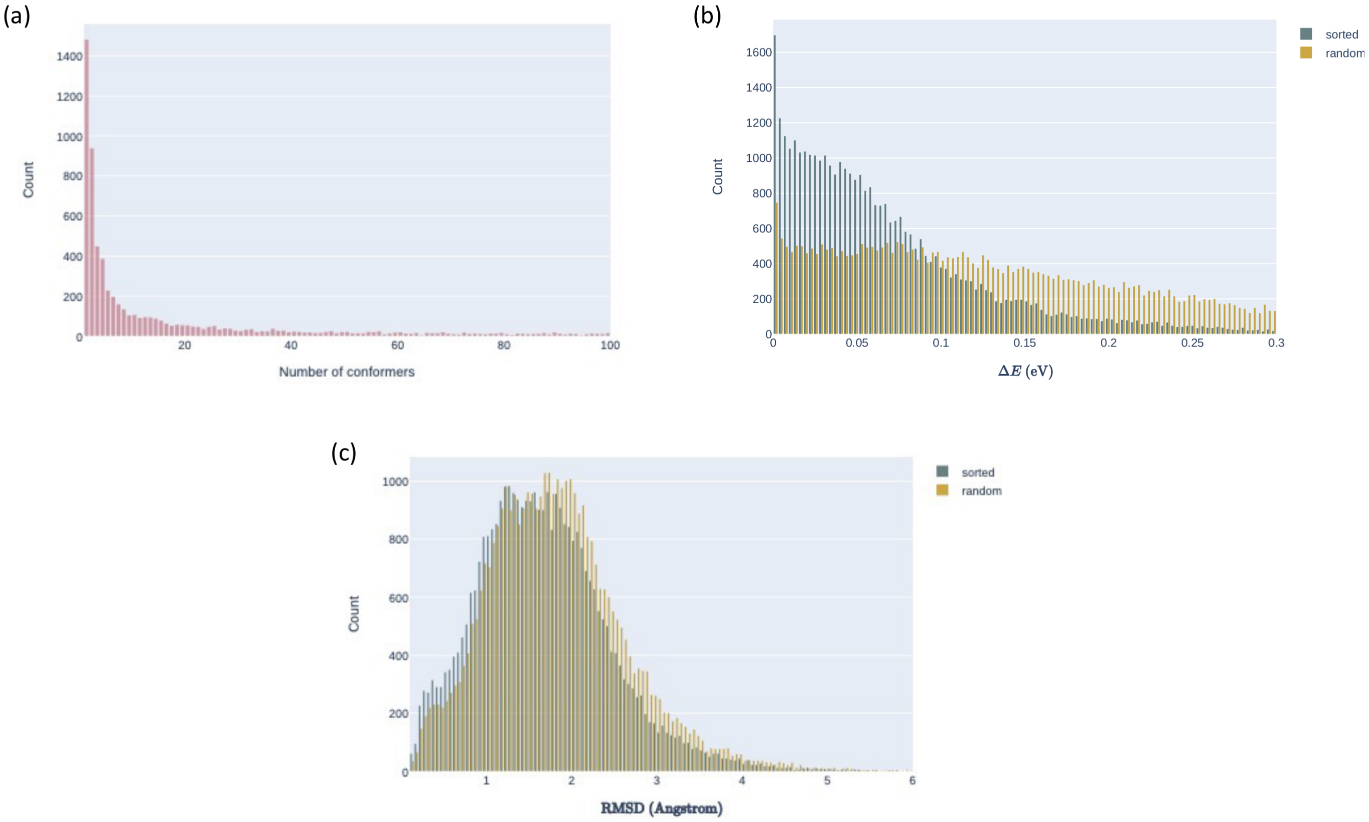

Figure S2 Ames dataset. Distribution of the (a) number of conformers, (b) energy difference between selected conformers referenced to the lowest energy conformer, and (c) RMSD between selected conformers referenced to the lowest energy conformer. Ten conformers were either selected by energy (sorted) or randomly (random).

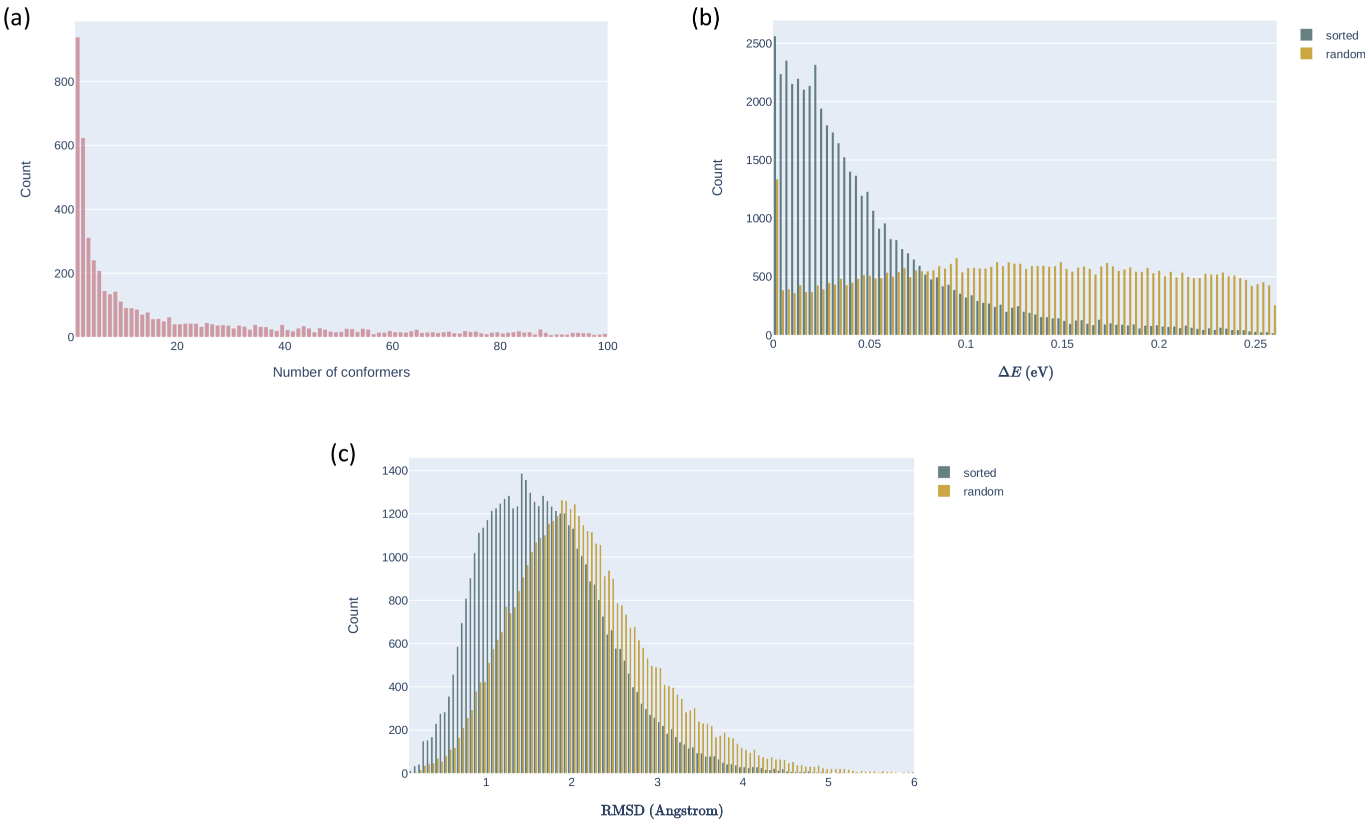

Figure S3 Tox21 dataset. Distribution of the (a) number of conformers, (b) energy difference between selected conformers referenced to the lowest energy conformer, and (c) RMSD between selected conformers referenced to the lowest energy conformer. Ten conformers were either selected by energy (sorted) or randomly (random).

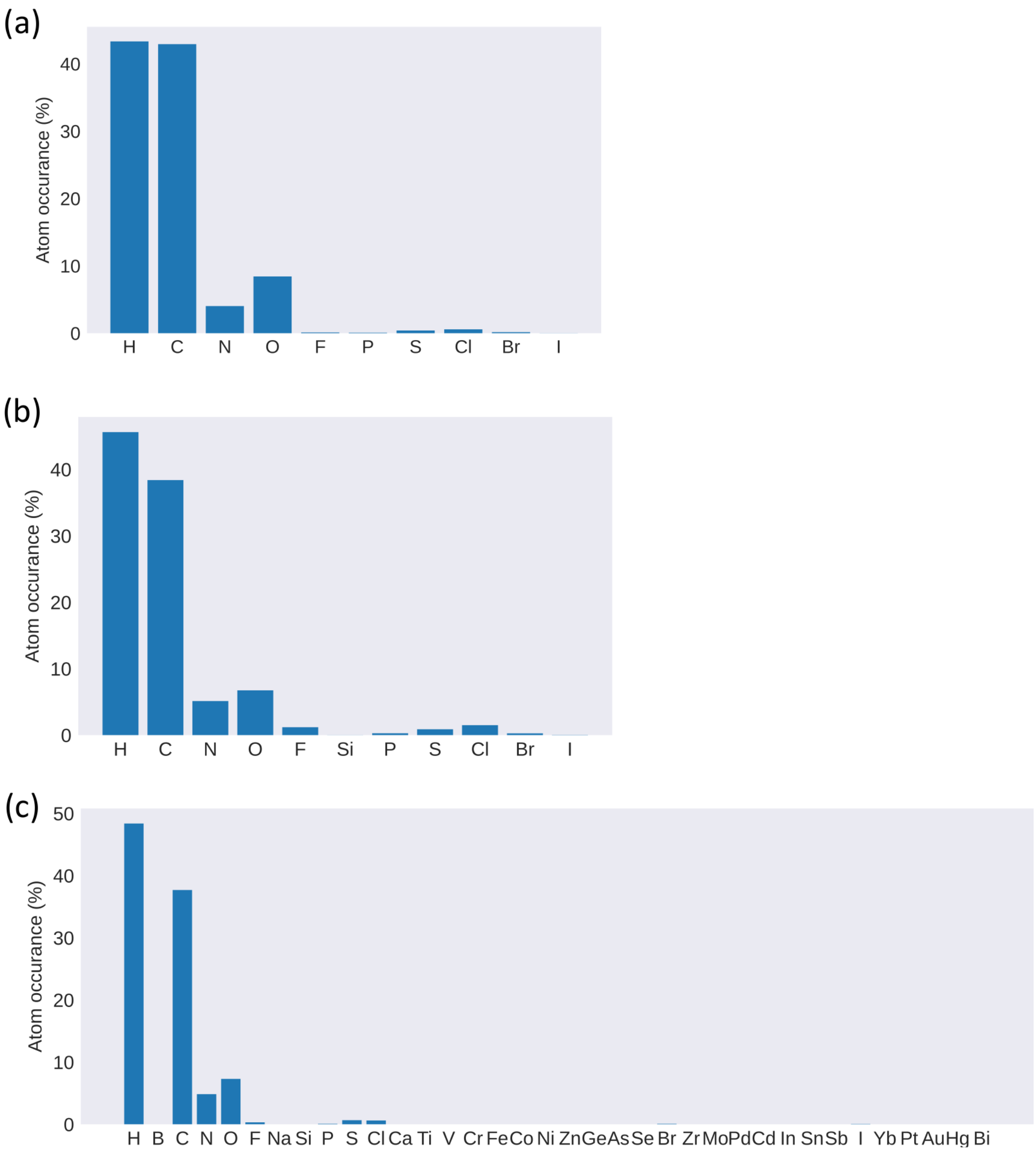

Figure S4 Distribution of atom types for the (a) Ames, (b) LD50, and (c) Tox21 datasets.

5 Attention weights statistics

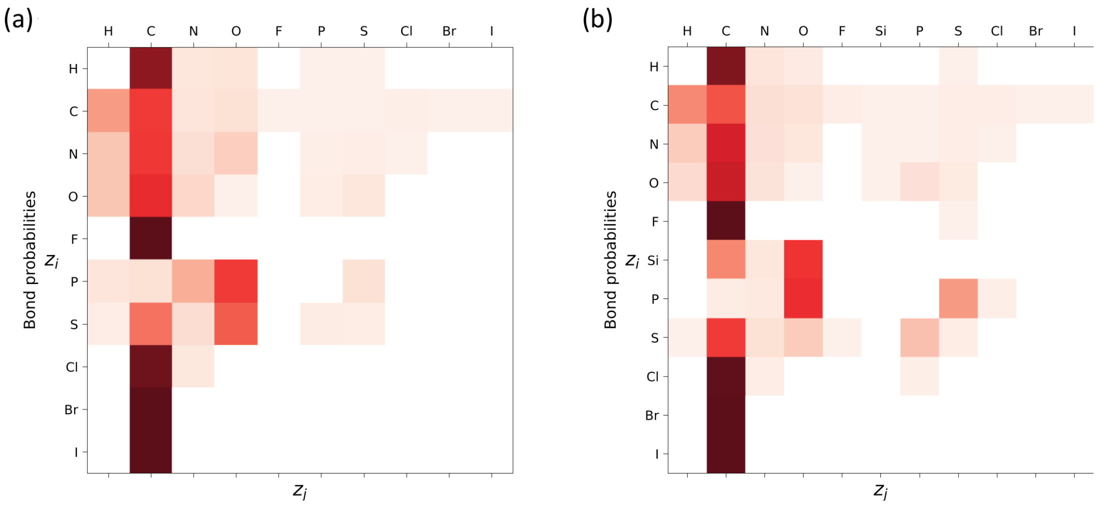

Figure S5 Visualization of bond probabilities evaluated on the (a) Ames and (b) LD50 datasets.

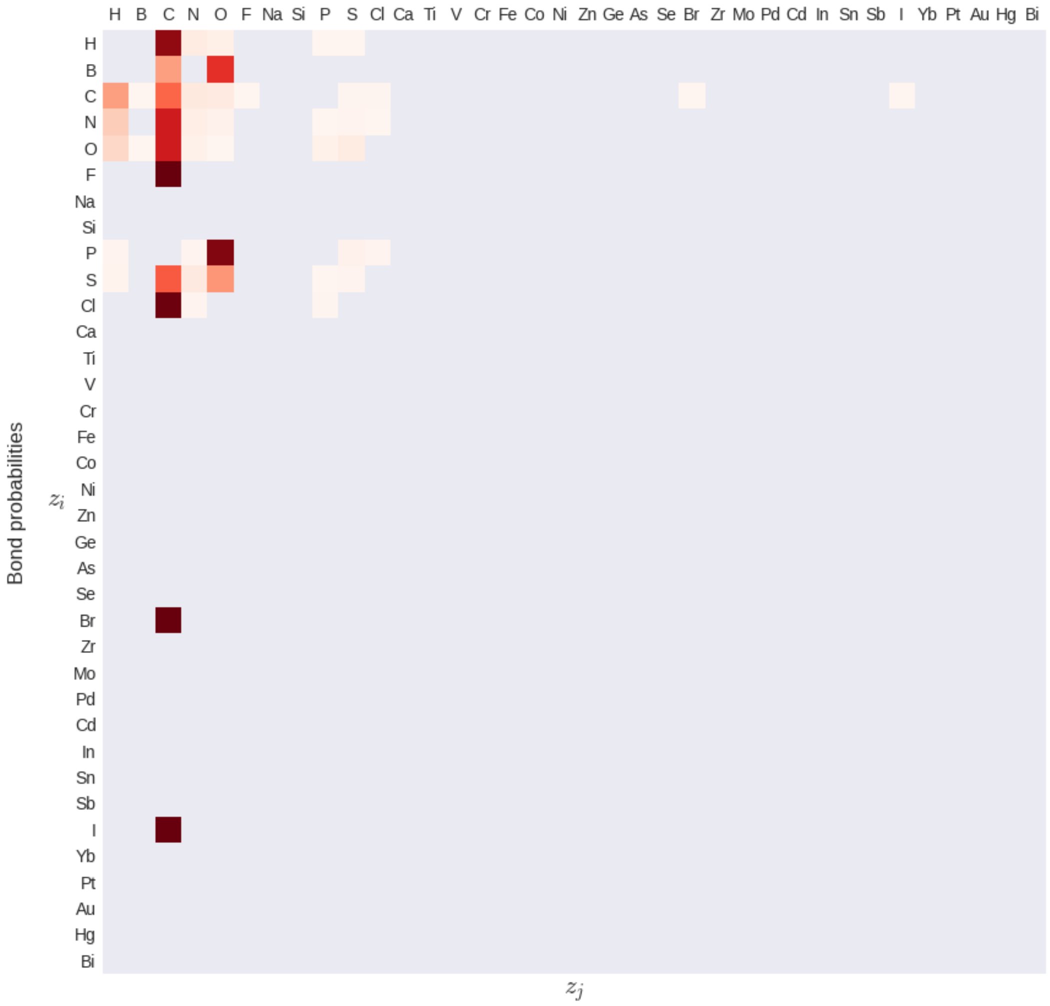

Figure S6 Heat map for the bond probabilities on the Tox21 test set. Atom z_i is attending to z_j.

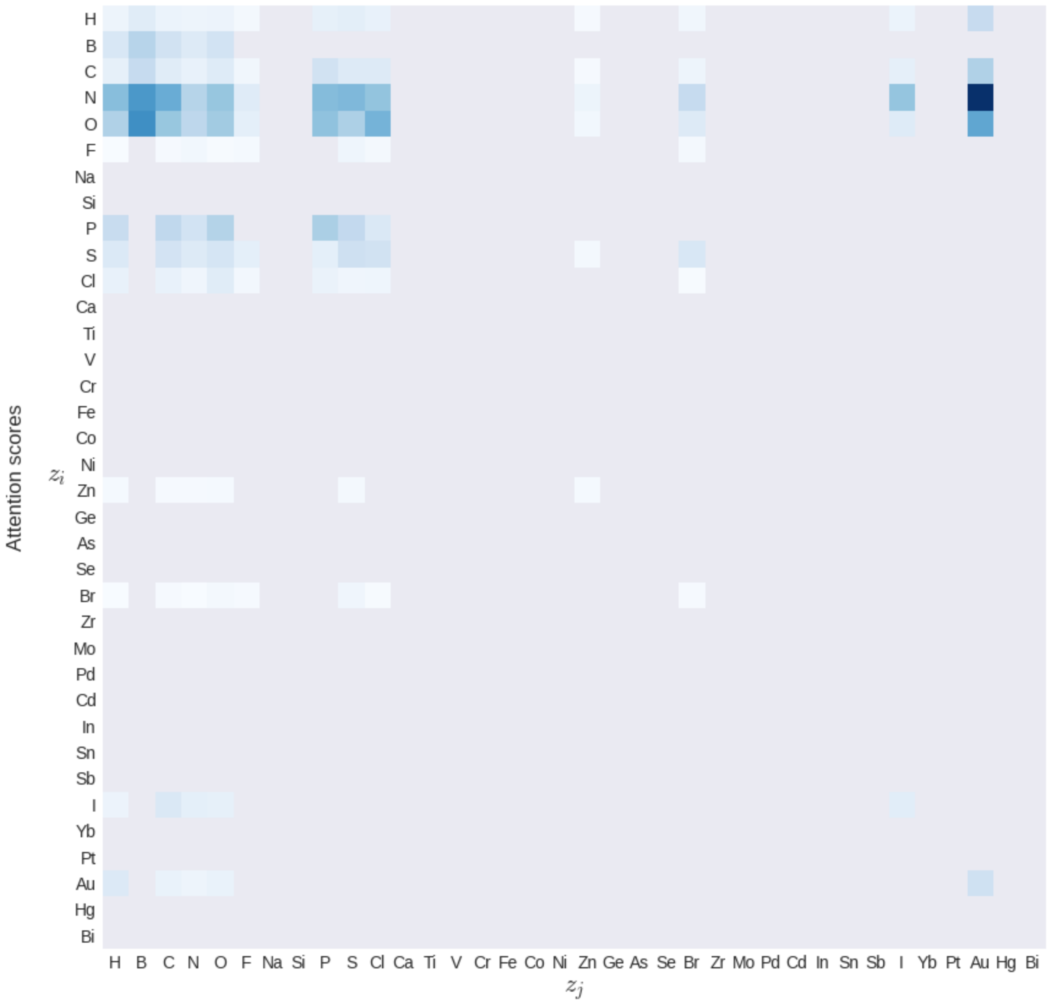

Figure S7 Heat map for the attention scores evaluated on the Tox21 test set. It shows the importance the model learned to give to atom z_i attending to z_j.
